# Point-of-care transcranial Doppler by intensivists

**DOI:** 10.1186/s13089-017-0077-9

**Published:** 2017-10-13

**Authors:** Vincent Issac Lau, Robert Thomas Arntfield

**Affiliations:** 1 Department of Medicine, Division of Critical Care, Schulich School of Medicine and Dentistry, Western University, London, ON Canada; 2 London Health Sciences Centre, Victoria Hospital Rm, D2-528, 800 Commissioners Road East, London, ON N6A 5W9 Canada

**Keywords:** Point-of-care ultrasound, Midline shift, Vasospasm, Intra-cranial pressure, Cerebral circulatory arrest, Neuro-critical care

## Abstract

In the unconscious patient, there is a diagnostic void between the neurologic physical exam, and more invasive, costly and potentially harmful investigations. Transcranial color-coded sonography and two-dimensional transcranial Doppler imaging of the brain have the potential to be a middle ground to bridge this gap for certain diagnoses. With the increasing availability of point-of-care ultrasound devices, coupled with the need for rapid diagnosis of deteriorating neurologic patients, intensivists may be trained to perform point-of-care transcranial Doppler at the bedside. The feasibility and value of this technique in the intensive care unit to help rule-in specific intra-cranial pathologies will form the focus of this article. The proposed scope for point-of-care transcranial Doppler for the intensivist will be put forth and illustrated using four representative cases: presence of midline shift, vasospasm, raised intra-cranial pressure, and progression of cerebral circulatory arrest. We will review the technical details, including methods of image acquisition and interpretation. Common pitfalls and limitations of point-of-care transcranial Doppler will also be reviewed, as they must be understood for accurate diagnoses during interpretation, as well as the drawbacks and inadequacies of the modality in general.

**Electronic supplementary material:**

The online version of this article (doi:10.1186/s13089-017-0077-9) contains supplementary material, which is available to authorized users.

## Background

In the absence of consciousness, a complete neurological exam is not always possible. Limited to the assessment of brainstem structures for neurological function (pupils, Glasgow Coma Scale, Cushing’s reflex, cranial nerve reflexes/responses and respiratory pattern), the intensivist at the bedside must instead rely on other tools to characterize sub-catastrophic anatomic events, as some of the above findings can be non-specific for the etiology of decreased level of consciousness. Most typically, this involves the use of computed tomography (CT) or magnetic resonance (MR) imaging, and in many cases, an invasive intra-cranial pressure (ICP) monitoring through insertion of an intracerebral catheter. Though these tools offer great value, they also introduce well-defined risks of transport or radiation [1, 2], and risk of an invasive procedure [3, 4], as well as delays in identifying and managing time-sensitive neurologic processes [5]. A more immediate, non-invasive, bedside approach to complement these existing methods is therefore of interest.

In 1982, Aaslid et al. introduced into clinical practice the use of transcranial Doppler (TCD) ultrasonography. TCD offers a non-invasive means of evaluating intra-cranial blood vessel flow and velocity with color and spectral Doppler [6]. The utility is evident, as it remains a cornerstone for neurologists, neurosurgeons, and intensivists in the identification of vasospasm after sub-arachnoid hemorrhage [7–9]. TCD may also be used to roughly predict ICP [10–14] as well characterize the alterations in blood flow that occur during intra-cranial cerebral circulatory arrest from severely raised ICP [15], although TCD cannot be used as a surrogate for invasive ICP monitor [16, 17].

As ultrasound technology has improved, the same transcranial acoustic windows used for the Doppler assessment of the cerebral circulation may also be used to achieve two-dimensional (2D) images of the brain parenchyma. Though anatomic detail is inferior to CT imaging, resolution is sufficient to answer emergent bedside questions such as mass effect leading to midline shift [18–20] and predict adverse outcomes after stroke [21, 22]. Given phenomena such as elevated ICP, vasospasm, midline shift, and brain death are of routine concern in the intensive care unit (ICU), it is appealing to consider this skill in the hands of the intensivist at the bedside for the expedition of care. Similar to all other point-of-care (POC) ultrasound applications, immediate, round-the-clock availability and repeatability in response to interventions or clinical change are particular advantages of TCD being in the hands of the intensivist. Additionally, resource-limited settings (remote areas, low-income countries, combat field, sports arenas, etc.) where CT access is scarce may be of particular benefit to acute care providers.

Despite the widespread availability of portable ultrasound machines in the ICU (due largely to the significant uptake of point-of-care echocardiography and critical care ultrasound in the critical care community), uptake of point-of-care TCD has not been observed. In the face of barriers to uptake such as the omission of TCD from the internationally adopted critical care ultrasound competency statement [23], it is important to illustrate the potential value and influence that point-of-care TCD may yield in the hands of the intensivist. To this end, our review will highlight potential applications of point-of-care TCD and how it may be used to screen four specific entities. Through four representative cases, the reader will be briefed on the theoretical and technical aspects of intensivist-conducted TCD for expedition of care. We will also describe the common pitfalls and limitations that are common to the technique.

## Example case descriptions

All figures/tables associated with the following cases in this article are original submissions.

### Case 1: midline shift

**Fig. 1:**
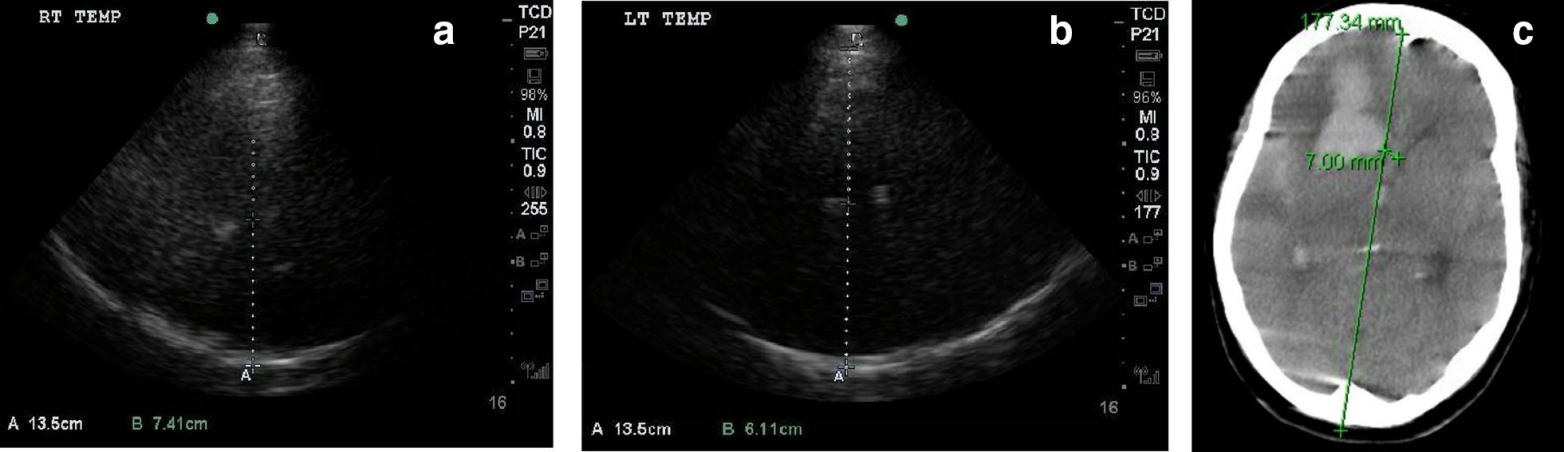
Transcranial imaging for midline shift. **a** Insonation from right temporal bone to third ventricle, representing distance A (7.41 cm). **b** Insonation from left temporal bone to third ventricle, representing distance B (6.11 cm). **c** Follow-up CT scan post TCD which reveals midline shift to be 7 mm

A 58-year-old female presented with a headache and decreased level of consciousness (LOC), and is found to have diffuse SAH, intra-cranial hemorrhage (ICH), and intra-ventricular hemorrhage (IVH) from a right middle cerebral artery (MCA) aneurysm with only 2 mm of initial midline shift measured by CT head. The patient had further deterioration in her neurological status with concerns of about increasing mass effect from her hemorrhages, and a point-of-care (POC) TCD 2D image was obtained (Fig. 1a, b), which found that her midline shift had increased to 6.5 mm towards the left:

$${\text{Midline shift }}\left( {\text{MLS}} \right) = \left( {{\text{distance A}} - {\text{distance B}}} \right)/ 2$$

$${\text{Midline shift }}\left( {\text{MLS}} \right) = ( 7. 4 1- 6. 1 1 \;{\text{cm}})/ 2 = 1. 3 \;{\text{cm}}/ 2 = 0. 6 5 \;{\text{cm}} = 6. 5 \;{\text{mm}}$$

This was later confirmed on urgent repeat CT head imaging (Fig. 1c) of 7 mm towards the left secondary to enlarging ICH.

### Case 2: vasospasm

**Fig. 2:**
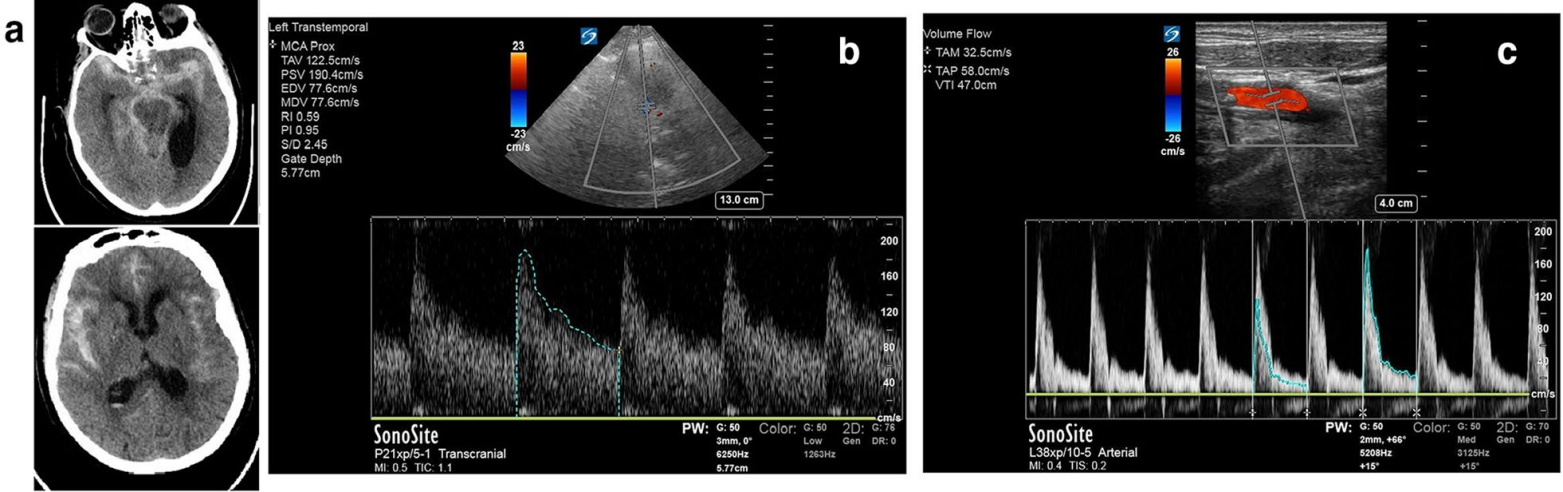
Transcranial Doppler for vasospasm following subarachnoid hemorrhage. **a** CT Head demonstrating diffuse subarachnoid hemorrhage. **b** TCD measurement of left MCA flows, demonstrating a mean MCA velocity of 123 cm/s (in-keeping with mild vasospasm). **c** Measurement of ipsilateral left ICA flows for calculation of Lindegaard ratio of 3.8 (Mean MCA/ICA velocity = 123/32.5 cm/s), which confirmed mild MCA vasospasm

An 83-year-old male with diffuse SAH from a right anterior-communicating (AComm) artery aneurysm (Fig. 2a), secured by coiling. Despite prophylactic nimodipine to improve outcomes in vasospasm patients, 5 days later, patient again had decreased LOC and required intubation. An urgent CT head was arranged, and an urgent bedside POC TCD (Fig. 2b) was rapidly performed, showing a mean velocity of 123 cm/s, with a Lindegaard ratio of 3.8 (Fig. 2c) which correspond with TCD signs of mild vasospasm (Additional file 1: Video 1). CT-angiogram confirmed this, including moderate vasospasm in the anterior cerebral artery (ACA) and posterior cerebral artery (PCA) territories. This prompted hypertensive therapy with vasopressors as per local neurosurgical standard of care. Follow-up serial screening TCDs and CT-As by radiology showed worsening to severe vasospasm in all vascular territories, first diagnosed on POC TCD.

### Case 3: raised intra-cranial pressure

**Fig. 3:**
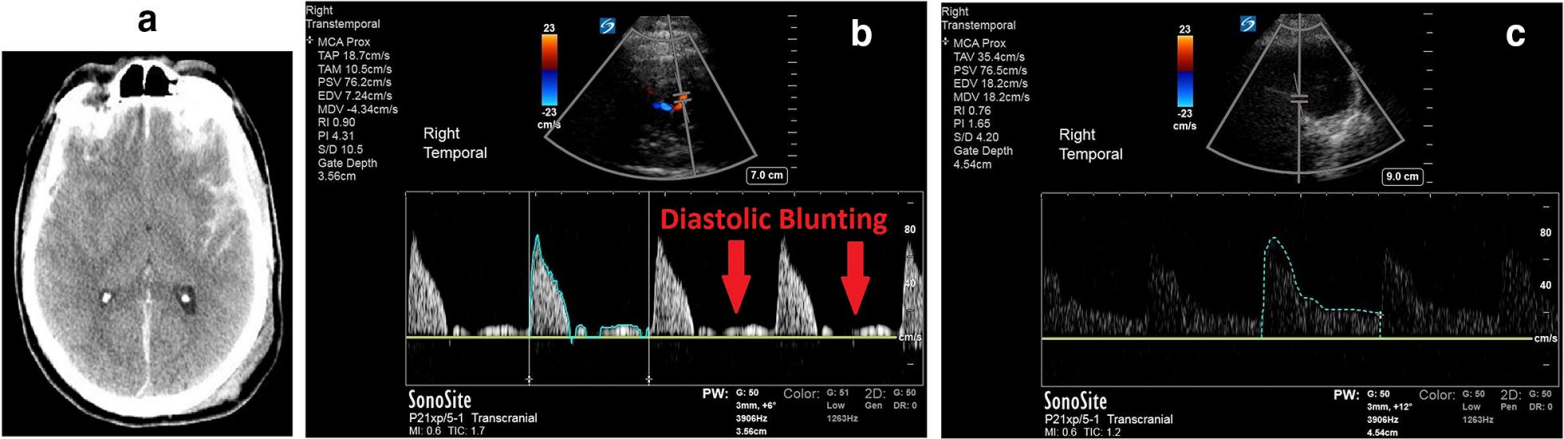
Raised intra-cranial pressure by spectral Doppler on TCD, as calculated by pulsatility index. **a** Diffuse subarachnoid hemorrhage on CT head. **b** Spectral Doppler of MCA, demonstrating diastolic blunting secondary to raised ICP (pulsatility index = 4.31, ICP = 46 mmHg). **c** Following interventions to reduce ICP, there was normalization of diastolic flow in the MCA, and resolution of high ICP (pulsatility index = 1.65, ICP = 17 mmHg)

A 74-year-old male, previously on dual antiplatelet agents (aspirin, clopidogrel) for cardiac stents, fell down 15 stairs. He was diagnosed with traumatic brain injury (TBI) on CT head (Fig. 3a) with diffuse sub-SAH and subdural hemorrhage (SDH), with an admission GCS of 14. GCS rapidly deteriorated to three, and a POC TCD was performed immediately (Additional file 2: Video 2) as the neurosurgeon was already preparing to insert an EVD following intubation. TCD was performed simultaneously while scalp was prepped on contralateral side. Interrogation of the MCA flows (Fig. 3b) showed a pulsatility index of 4.31 (equating to an ICP of 46 mmHg by TCD). The extra-ventricular drain (EVD) was placed at the bedside for therapeutic effect and confirmed the TCD findings of ICP elevation (EVD monitor measured ICP = 42 mmHg). After appropriate neuro-resuscitative measures (osmotic therapy, vasoactive agents and ICP drainage via EVD), a follow-up POC TCD (Fig. 3c) showed decreased ICP (TCD pulsatility index = 1.65, ICP = 17 mmHg). These dynamic changes were also mirrored in agreement with the EVD monitor (EVD ICP = 16 mmHg).

### Case 4: cerebral circulatory arrest

**Fig. 4:**
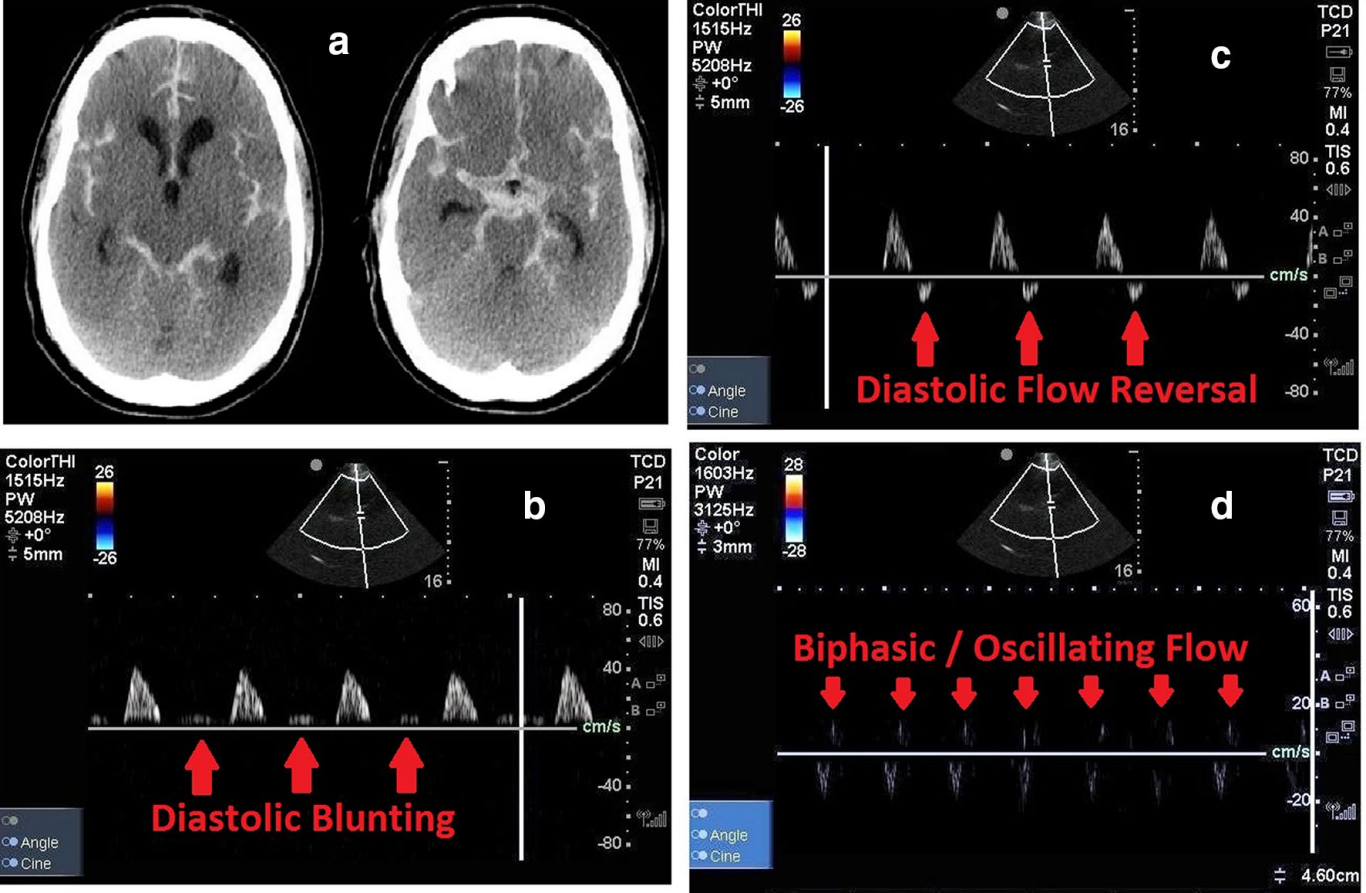
Step-wise progression of cerebral circulatory arrest. **a** CT Head demonstrating diffuse subarachnoid hemorrhage. **b** Evidence of raised ICP causing decreased diastolic flow as evidenced by blunting of the spectral Doppler signal. **c** Further progression with diastolic flow reversal as raised ICP prevents forward flow in MCA, and even induces backwards flow. **d** Biphasic and oscillating flow as evidenced by net zero flow (where systolic and diastolic flow are near equal to each other), indicating the first TCD stage of cerebral circulatory arrest

A 63-year-old male presented with retro-orbital headaches was diagnosed with an AComm artery aneurysm which underwent a coiling attempt. During the procedure, however, the patient had internal carotid artery (ICA) dissection, trans-mural perforation and pseudo-aneurysm formation. CT head revealed diffuse SAH from aneurysmal rupture with IVH (Fig. 4a). An EVD was placed immediately at bedside, which showed raised ICP (> 60 mmHg). A poor prognosis was given and goals of care changed to Do Not Resuscitate (DNR). The patient subsequently developed dilated pupils bilaterally and had hemodynamic alterations in keeping with a Cushing’s response. A POC TCD was obtained serially to determine and characterize the progression to brain death through the predictable spectral Doppler evolution: diastolic blunting, diastolic flow reversal, and, finally, biphasic/oscillating flow (Fig. 4b–d).

## Methods

### Ultrasound technique

**Fig. 5:**
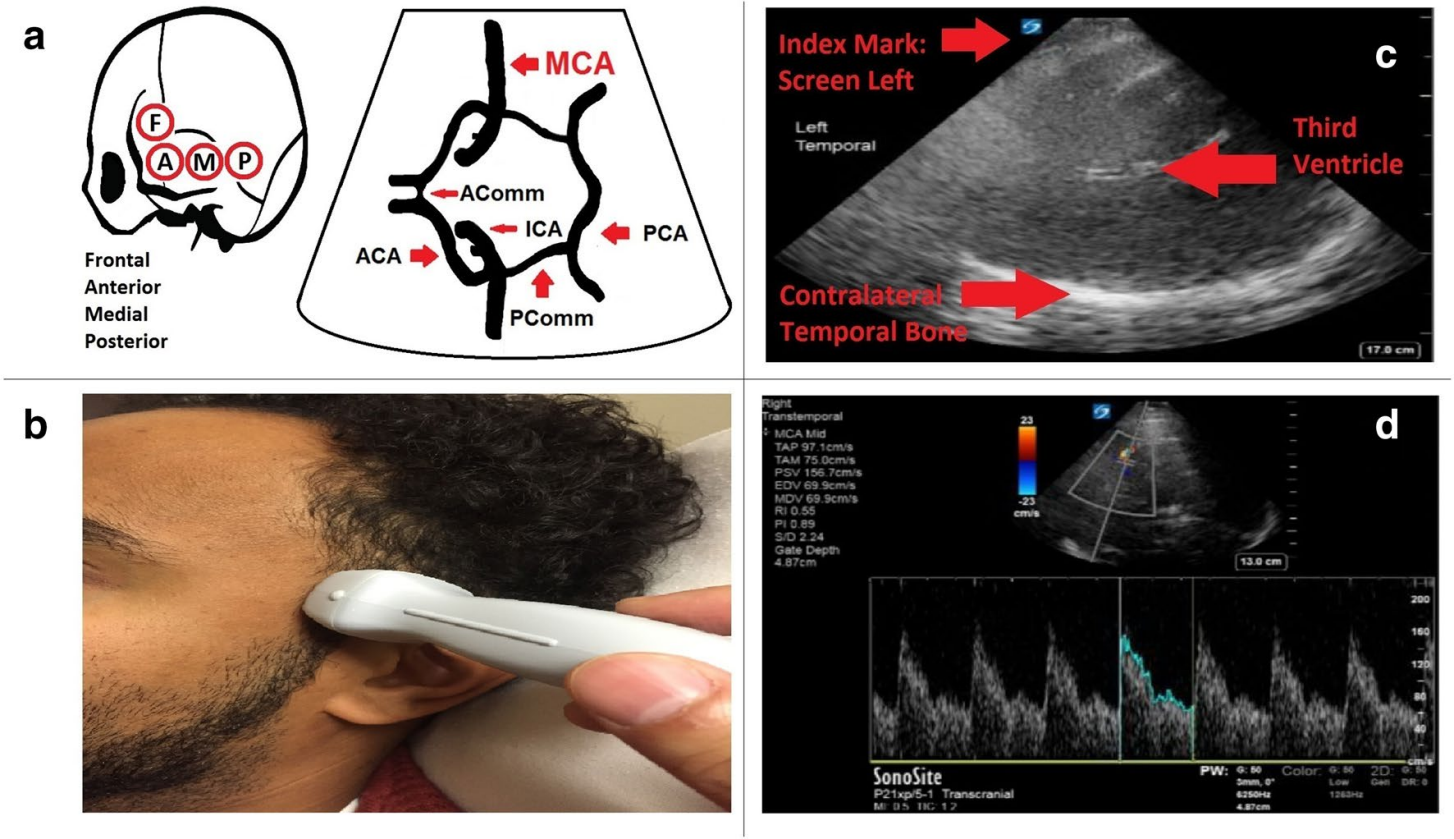
Method for acquisition of transcranial Doppler. **a** Left side shows location of the trans-temporal window and its various sections: frontal, anterior, medial, and posterior. Right side illustrates Circle of Willis, with MCA highlighted. **b** Probe index mark orientation towards the patient’s anterior/front. **c** Typical 2D image of TCD from trans-temporal window with index mark to screen left, with bright echogenic contralateral temporal bone, and anechoic space of midline third ventricle. **d** Typical spectral Doppler velocity waveform from MCA, with steep systolic upstroke and step-down diastolic flow (typical mean velocity of 80 cm/s)

To perform a TCD or TCD image (TCDI), place your patient in the supine position with the head of bed > 30° (not always possible in ICU patients). Use a 1–5 MHz phased array ultrasound transducer with a TCD preset, or if unavailable, a cardiac preset. For either preset, the sonographer must ensure that the lowest Nyquist level should be selected (~ 20 cm/s) for color-coded duplex sonography (CDDS). Place the probe on the trans-temporal window (Fig. 5a), with the index mark pointed towards the patient’s anterior/front (Fig. 5b). With the index mark orientated to screen left, identify the ipsilateral/contralateral temporal bones, and the third ventricle (a midline structure) (Fig. 5c). Decrease the depth to the distance of the third ventricle in the far-field and identify the cerebral penducles and echogenic basal cisterns. Then place a color Doppler box over the top half of the screen (near field) where the MCA is located, just lateral to the cerebral penducles. Identify the MCA as indicated by red color Doppler signal towards the probe. Then interrogate the MCA by placing the pulse wave marker on top of the MCA, and obtain the spectral Doppler waveforms (Fig. 5d). Angle correction should be utilized upon the insonation of vessels in order to adjust the pulse wave Doppler for the angle of insonation.

MCA interrogation should be obtained at 0.5 cm for the M1 segment, and 0.4 cm for the M2 segment of the MCA. Intervals at its most distal point to the bifurcation of the proximal ICA into the MCA and ACA, to screen for focal vasospasm. The trans-temporal window can also insonate the ACAs (anterior angulation, depth 6–7 cm), terminal ICAs (caudal angulation, 6–7 cm), and PCAs (posterior angulation, 5.5–7.5 cm), bilaterally. The protocol is repeated for the opposite hemisphere. Trans-orbital and trans-foraminal windows are more typical for complete diagnostic TCD, and will not be discussed as part of POC TCD.

## Discussion

This review seeks to characterize what we feel are the four major point-of-care applications for TCD in the hands of critical care providers. There are many more additional potential indications and applications for the use of TCD at the point-of-care (full list shown in Table 1). However, outside of the four chosen POC TCD indications, the remainder are the scope of formal TCD, as they require additional training [11, 12, 18, 22, 24–32].

**Table 1:** Indications for transcranial ultrasonography and Doppler

Indication
Midline shift
Vasospasm (post-subarachnoid hemorrhage)
Intra-cranial pressure (ICP)
Cerebral circulatory arrest (brain death)
Stenosis
Occlusion/stroke
Micro-emboli (transient ischemic attack or stroke)
Right-to-left shunts (paradoxical emboli)
Sickle cell hyperemia versus stenosis
Intra-cranial hemorrhage detection and monitoring
Cerebral motor vasoactivity
A–V malformation assessments
Syncope or positional vertigo

There are several advantages to use of transcranial imaging and Doppler. It is quick, with no radiation exposure (unlike CT), and requires no patient transport (unlike MRI or CT). Additionally, unlike traditional 4-vessel angiography, TCD is a non-invasive means of monitoring for vasospasm, stenosis, stroke, ICP or cerebral circulatory arrest [18, 19, 24]. Accuracy of point-of-care TCD is unknown, the diagnostic sensitivity of TCD in the hands of expert sonographers is quite good, reaching 89–98% for the MCA [18, 19].

The four themes will be further characterized here with a focus on the technical elements as well as the value of rapid application at the bedside. As with any ultrasound modality, the acquisition of images and Doppler information is operator-dependent, and there are several limitations and pitfalls to TCD acquisition and interpretation, discussed within each topic.

## Midline shift

Diagnosis of midline shift is important both for preventing further secondary neurological injury by early neurosurgical intervention, but also neuro-prognostication. Any amount of midline shift is considered abnormal, but poor neurological outcome can be associated with a clinically significant midline shift of as little as 0.5 cm (positive predictive value (PPV) of 78% with presence of midline shift [20, 25], as opposed to a 14% PPV without the presence of midline shift), and a twofold increase in mortality associated with greater than 1-cm midline shift (53% vs 25%) [21, 22].

Seidel et al. illustrated the use of ultrasonography for the measurement of midline shift. Reproducibility of MLS via ultrasound corresponded to 0.3 ± 0.2 mm in ten volunteers [20]. Measurements on ultrasound have correlated well with CT findings [20–22], and have been predictive of poor outcome from midline shift secondary to pathologies such as stroke, hemorrhage (subdural, epidural, subarachnoid), and traumatic brain injury [20–22, 25, 26].

There are two methods to quantify midline shift. One method (see Fig. 1a, b) is to measure the distance from the bilateral temporal bones to the midline third ventricle. Distance A is measured from the ipsilateral side, whereas distance B is from the contralateral side. One should also measure the full length from the ipsilateral to the contralateral temporal bone. Then use the following equation to calculate the midline shift deviation:

$${\text{Midline shift }}\left( {\text{MLS}} \right) = \left( {{\text{distance A}} - {\text{distance B}}} \right)/ 2$$

For an expedited investigation, the other method (see Fig. 1a, b) would be to measure from the ipsilateral side to the third ventricle only (distance C), and then also measure the full distance from the ipsilateral temporal bone to the contralateral temporal bone (distance D), then calculate MLS with this equation:

$${\text{Midline shift }}\left( {\text{MLS}} \right) = {\text{distance C}} - \left( {{\text{distance D}}/ 2} \right)$$

With either method, if the MLS is positive, this means that the MLS is away from the ipsilateral side. If the MLS is negative, the MLS is toward the ipsilateral side.

Our recommendation is to utilize the first method for midline shift quantification. With 2 independent measurements from each side to assess midline shift and an internal check for correct measurements (the sum of distance A and B should equal the full distance from the ipsilateral to contralateral temporal bones), it is the least vulnerable to operator error. Clinical utilization of TCD or TCDI for midline shift also incorporates comparisons of measured cerebral blood flow velocities and pulsatility indexes between right and left anterior circulation. This will be discussed in the vasospasm and raised ICP sections, as discrepant findings and asymmetry in CBFs and PIs are a main indicator of potential midline shift.

### Midline shift: limitations

Due to thicker cranial vaults causing higher bone attenuation, the literature states that 5–20% of patients will have difficult views leading to un-interpretable transcranial windows and images [18, 19]. MLS measurement relies heavily on finding a proper trans-temporal window. There are no data correlating angle of insonation and accuracy of transcranial US midline shift measurements. AIUM guidelines state that the upward angle of insonation should be no greater than 10–15° [7], but that may not always be possible. The presence of hydrocephalus of the third ventricle does not have much bearing on the MLS measurement, as the measurements are done to the center of the third ventricle, not to its outer walls. However, hydrocephalus does help predict the need for EVD insertion and for safe EVD removal. Kiphuth et al. observed that TCD was a reliable method for predicting the necessity for CSF drainage. In patients with external ventricular drainage (EVD), they estimated that a cut-off value of an increase of 5.5 mm in ventricle width after clamping had a high sensitivity (100%) and negative predictive value (100%). They suggested that an increase in ventricular width lower than the cut-off was an indication for a safe removal of EVD [27].

## Vasospasm

Transcranial Doppler has been studied extensively as a validated screening tool for diagnosing vasospasm [6, 7, 28–41], aiding in the management of subarachnoid hemorrhage (SAH) patients. Diagnosis of vasospasm post-SAH greatly affects prognosis, causing significant morbidity secondary to strokes from delayed cerebral ischemia, and mortality in 15–20% of patients [8, 37, 39]. Using the inverse relationship between cerebral blood vessel diameter and TCD mean velocities, we are able to quantify and subcategorize vasospasm [25, 35, 36].

Assessment of the MCA requires pulse wave spectral Doppler. The normal spectral Doppler profile of the MCA has a sharp systolic upstroke with a step-wise diastolic deceleration, with a normal mean velocity of the MCA that is usually < 80 cm/s. Mild vasospasm mean velocities are 120–159 cm/s, moderate vasospasm is 160–199 cm/s, and severe vasospasm is > 200 cm/s. These cutoffs have been derived using non-imaging traditional TCD probes as are commonly used in complete, diagnostic TCD [6, 9, 28, 36, 42]. Progressive increase of mean velocities during the early stages of SAH to predictive of vasospasm. Authors have cited a change in baseline mean velocity of > 21 cm/s per 24 h in the first 3 days to be diagnostic of vasospasm [39, 43]. Symptomatic clinical vasospasm is often only seen at mean velocities of 160 cm/s [38].

Another method of grading vasospasm severity would be the formula known as the Lindegaard ratio [36]:

$${\text{Lindegaard ratio}} = {\text{Ipsilateral MCA mean velocity}}/{\text{ipsilateral extra}} - {\text{Cranial ICA mean velocity}}.$$

As entities other than vasospasm (e.g., hyper-dynamic flow) may increase mean velocities, the Lindegaard ratio does not grade vasospasm, but helps differentiate between hyperemia (induced by medial or endovascular treatment) versus the onset of true vasospasm. If the Lindegaard ratio > 3, this would diagnose vasospasm, as increased flow velocity would be raised in the cerebral circulation relative to the carotid circulation. A Lindegaard ratio of 3–5 would denote mild-moderate vasospasm, while a ratio > 6 would indicate severe vasospasm [36].

### Vasospasm: limitations

The diagnostic value of TCD clinical utilization to detect vasospasm is not in question. All relevant professional societies, like American Heart Association, American Society of Neuroimaging, and American Institute for Ultrasound in Medicine all recommend TCD utilization for vasospasm [7, 44–46]. Compared to CT-angiogram, the sensitivity/specificity of the MCA is quite good (~ 89–98%). However, the trans-orbital and trans-foraminal windows are less reliably insonated compared to the trans-temporal window [47], and the ACA and PCA are less sensitive and specific for vasospasm compared to the MCA. The same applies to the basilar artery and vertebral artery with lower sensitivity and specificity in the trans-foraminal window [30, 31]. Exhaustive exclusion of vasospasm calls for all cerebral vessels to be insonated. Thus, the technique as we have described it represents a screening test of the highest yield arterial territory (MCA)—of greatest value if vasospasm is identified and insufficient to exclude the possibility [6, 42].

Several factors make the diagnosis of vasospasm challenging. The clinician must be mindful that cerebral blood flow maybe influenced by many factors (PaO_2_, PaCO_2_, blood viscosity, collateral flow). Furthermore, operator experience is required for accurate assessment, as improper vessel identification, proximal lesions and aberrant vessel course can confound even experienced sonographers [9].

For Doppler interrogation, diagnostic TCD utilizes non-imaging probes set for measurement of spectral Doppler signs at specific distances to insonate various vessels in question. However, most radiology departments now use color-coded duplex sonography (CDDS) imaging probes with pulse wave Doppler and angle correction [48]. The advantage to using CDDS imaging probes is that insonation no longer needs a perfectly on-axis due to anatomic variations between different segments of cerebral arteries, as machines are able to solve for off-axis measurements using angle correction. The caveat is that normal velocities reported for TCD are validated for non-imaging probes and not for color-coded duplex sonography [48]. However, many radiology departments utilize CDDS instead of traditional non-imaging TCD probes despite the lack of validation prior. Absolute velocities obtained from CDDS would be underestimated, but CDDS angle correction has been shown to be no different than velocities obtained from traditional TCD [49].

## Intra-cranial pressure

**Fig. 6:**
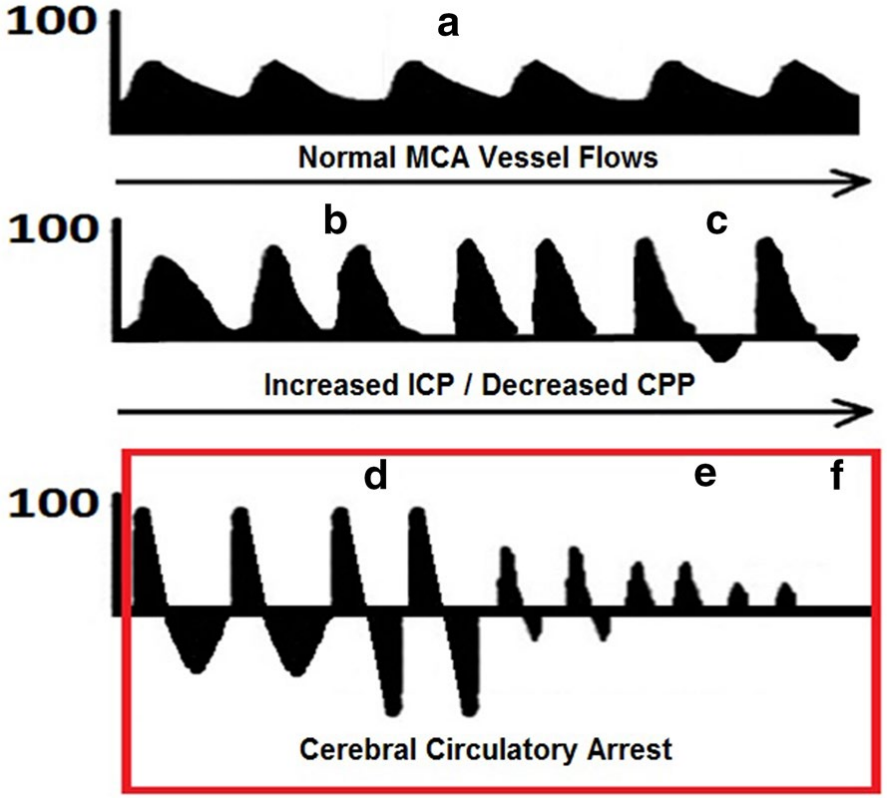
Progression of intra-cranial circulatory arrest via transcranial Doppler of middle cerebral artery flows. **a** Normal systolic upstroke with normal step-down of diastolic flow. **b** Increased peak systolic flow with decreasing diastolic flow and eventual blunting of diastolic flow. **c** Diastolic flow reversal. **d** Biphasic or oscillating flow—where diastolic flow reversal approaches equal size to systolic flow. **e** Isolated sharp systolic peak flows of < 200 ms and small systolic amplitude of < 50 cm/s. **f** Zero flow—where there was previously documented TCD flow. The red box denotes states (**d**, **e**, **f**) in which cerebral circulatory arrest can be diagnosed

TCD can be used to give a rough estimate for ICP, to help rule-in high ICP, but not as a surrogate for accurate invasive ICP monitors. As ICP increases, flow in intra-cranial vessels changes. Initially, systolic velocity increases (i.e., systolic peak flows) as increased ICP causes cerebral vessels to narrow from external pressure in the MCA. During diastole, diastolic flow becomes decreased/blunted (Fig. 3b), as raised ICP becomes the predominant external pressure opposing forward MCA flow during diastole. Raised ICP can also exceed normal forward flow during diastole, leading to diastolic flow reversal (Fig. 6) [10, 15].

Raised ICP can be estimated using the Gosling’s pulsatility index, which is a reflection of peripheral resistance, which is equal to the difference between the peak systolic velocity (PSV) and end-diastolic velocity (ESV), divided by the mean velocity (MV) [10, 49–51]:

$${\text{Pulsatility index }}\left( {\text{PI}} \right) = \left[ {{\text{PSV}} - {\text{EDV}}} \right]/{\text{MV}}.$$

Bellner et al. correlated pulsatility index (PI) to ICP in clinical practice. Generally, high ICPs result in high pulsatility indices (PIs) in high-resistance vascular beds, where systolic velocity is increased relative to decreased end-diastolic velocity. This translates to an increased systolic-to-diastolic (S/D) ratio and an increased pulsatility index. A formula has been derived to convert pulsatility index into ICP (from all causes), with a sensitivity of 89%, and specificity of 92% [10].

$${\text{ICP}} = ( 10. 9 3 \times {\text{PI}}) - 1. 2 8.$$

Based on this formula, a PI of > 2.13 would correlate to an ICP > 22 mmHg (based on new Brain Trauma Foundation guidelines cutoffs) [52], which is the clinically significant cutoff for raised ICP, and would compromise cerebral perfusion pressure (CPP) [10, 53–55], whereas normal pulsatility index (PI) is < 1.2, and corresponds to an ICP of approximately 12 mmHg (normal ICP = 5–15 mmHg) [28]. The main advantage of PI is that it is not affected by the angle of insonation [18]. This is one of many TCD-derived PI formulas for estimation of ICP [12, 56–58]. Some suggest using TCD as a binary assessment for the absence of intra-cranial hypertension [12].

### Intra-cranial pressure: limitations

The main limitation of TCD PI correlation to ICP is that caution must be exercised given its wide confidence intervals when compared directly to ICP monitors [16, 17]. Other literature suggests that TCD PI is not a reliable correlate to ICP [13, 16, 17, 59–64]. Decreased PaCO_2_ or increased arterial blood pressure (ABP) alterations can influence cerebral blood flow and PI independently of ICP. Decreases in CPP (representing an increasing trend in PI) can be from increased ICP, but also decreases in mean arterial pressure (MAP). Therefore, instead of assuming linear correlation with ICP, PI can rather be understood to be inversely proportional to mean CPP or directly proportional to ABP, and a non-linear proportion to artery/arteriole compliance in the cerebral bed, cerebrovascular resistance, and heart rate. As such, recommendations for utilization of increasing PI for following increasing ICP and decreasing CPP trends across time, rather than ICP absolute values [59].

**Fig. 7:**
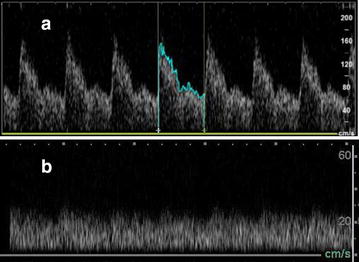
Disadvantage to transcranial Doppler when interpreting MCA flows for patient with non-pulsatile flow states (i.e., V–A ECMO, LVAD). **a** Normal pulsatile flow: systolic upstroke with normal step-down of diastolic flow. **b** Non-pulsatile flow: Loss of sharp systolic peak flows, with overall lowering of means flows—which would make interpretation of pulsatility index for ICP as well as mean flows for vasospasm more difficult

Although not a continuous means of ICP measurement, TCD PI can be performed non-invasively serially, helping to rule in high ICP, and prompting more invasive, continuous modalities. Confounders can make PI uninterpretable for raised ICP. Lack of pulsatile flow, such as with venous–arterial (V–A) extracorporeal membrane oxygenation (ECMO) or left ventricular assist devices (LVADs), as systolic and diastolic ratios for pulsatility index would be uninterpretable for ICP and brain death. The diagnosis of vasospasm by TCD would also be more difficult given the lower flow states associated with non-pulsatile flow from an LVAD or V–A ECMO (Fig. 7).

## Cerebral circulatory arrest

Predictable, step-wise changes in cerebral blood flow may be observed as part of progression to cerebral circulatory arrest (Fig. 6): decreasing or blunted diastolic flow, oscillating flow (characterized by diastolic flow reversal), sharp systolic peak flows, and then finally, zero flow in keeping with cerebral circulatory arrest [10, 15].

Serial interrogations are often required for TCD diagnosis of cerebral circulatory arrest and brain death, including a scan preceding the onset of cerebral circulatory arrest to demonstrate the presence of prior intra-cranial flow [15, 65–67]. Cerebral circulatory arrest may be identified on TCD (red box of Fig. 6) when one of three criteria are met in bilateral blood vessels, measured at least twice, 30 min apart: (1) Net-zero oscillating waveform flow with near equal systolic forward flow and diastolic reversed flow. (2) Small sharp systolic peaks with < 50 cm/s peak systolic velocity and < 200 ms duration. (3) Disappearance of all previously seen intra-cranial flow, but extra-cranial flow still present [30, 65]. These criteria (listed in Table 2) have a sensitivity of 88%, and specificity of 98% [30].

**Table 2:** Cerebral circulatory arrest criteria by transcranial Doppler

Oscillating wave form (equal systolic forward flow and diastolic reversed flow)
Small systolic spikes of < 200 ms duration and < 50 cm/s pulse systolic velocity spike
Disappearance of all intra-cranial flow (loss of systolic and diastolic flow, where flow had been present previously intra-cranially, and still present extra-cranially)

Determination of brain death is typically a clinical diagnosis with parameters like brainstem reflex testing and an apnea test. Certain situations (i.e., presence of spinal reflexes, drug ingestions, and profound hypothermia/shock) may lead to confounding with brain death determination if relying on clinical testing alone. If TCD rules in cerebral circulatory arrest by non-reassuring MCA flows, ancillary testing (4-vessel cerebral angiography, nuclear medicine radionuclide brain perfusion scan, CT or MR cerebral blood flow angiography) could be sought to formally confirm the diagnosis. However, if TCD shows reassuring cerebral blood flows not meeting aforementioned criteria, this would possibly save premature, unnecessary serial ancillary tests, until the diagnosis can be first confirmed on TCD. This helps optimizing the ideal time to obtain ancillary testing when clinical determination of brain death has confounders [65–70].

### Cerebral circulatory arrest: limitations

In addition to previously discussed limitations in other sections, there are specific limitations to TCD diagnosis of brain death. Despite the presence of reassuring spectral Doppler waveforms, a patient can still qualify as brain dead by other clinical means (brainstem and apnea testing in the absence of confounders). This means cerebral blood flow maybe inadequate to sustain life despite reassuring waveforms. The opposite is also true, as the absence of reassuring spectral Doppler waveforms (meeting above criteria) does not automatically mean brain death, as TCD evaluates cerebral circulatory arrest, not brainstem function. Furthermore, most medical jurisdictions will not accept a TCD as its own ancillary test to confirm brain death [67].

## Conclusions

The advent of intensivist-performed ultrasound and availability of ultrasound machines now provides a unique climate where point-of-care TCD is a reality. Intensivists with this skill are able to provide immediate, 24/7 bedside assessment for midline shift, elevated ICP, vasospasm and intra-cranial hypertension progression. Barriers to training do still exist, but despite these pitfalls and limitations, intensivists performing limited point-of-care TCD as a screening tool to rule-in certain indications is feasible. Reaching competence is attainable, without extensive formal radiology training for diagnostic TCD. Much like many other POC modalities, to rule in various pathologies where prompt bedside diagnosis could be invaluable to the expedition of patient care. We support the use of TCD as a complementary adjunct to routine investigations (formal TCD, CT/MRI, ICP monitors).

## Additional files

**Additional file 1: Video 1.** Vasospasm on TCD. Demonstration of vasospasm pulse wave Doppler of the MCA during TCD interrogation.

**Additional file 2: Video 2.** Raised ICP on TCD. Pulse wave Doppler of the MCA during TCD interrogation demonstrating raised ICP.

## Abbreviations

TCD: transcranial Doppler
CT: computerized tomography
CT-A: computerized tomography-angiogram
MRI: magnetic resonance imaging
MCA: middle cerebral artery
ICP: intra-cranial pressure
ICA: internal carotid artery
SAH: subarachnoid hemorrhage
IVH: intra-ventricular hemorrhage
ICH: intra-cranial hemorrhage
EVD: extra-ventricular drain
POC: point-of-care
GCS: Glasgow Coma Scale
TBI: traumatic brain injury
ICU: intensive care unit
PaCO_2_: pressure in arterial blood of carbon dioxide
PEA: pulseless electrical activity
CPR: cardio-pulmonary resuscitation
ROSC: return of spontaneous circulation
DNR: do not resuscitate
PPV: positive predictive value
MLS: midline shift
CPP: cerebral perfusion pressure
PI: pulsatility index
PSV: peak systolic velocity
ESV: end-diastolic velocity
MV: mean velocity
ABP: arterial blood pressure
MAP: mean arterial pressure
ONSD: optic nerve sheath diameter
V-A ECMO: venous-arterial extracorporeal membrane oxygenation
LVAD: left ventricular assist device
